# Genome overview of eight *Candida boidinii* strains isolated from human activities and wild environments

**DOI:** 10.1186/s40793-017-0281-z

**Published:** 2017-12-02

**Authors:** Salvatore Camiolo, Cinzia Porru, Antonio Benítez-Cabello, Francisco Rodríguez-Gómez, Beatríz Calero-Delgado, Andrea Porceddu, Marilena Budroni, Ilaria Mannazzu, Rufino Jiménez-Díaz, Francisco Noé Arroyo-López

**Affiliations:** 1 Dipartimento di Agraria, Università degli Studi di Sassari, Viale Italia 39, Sassari, Italy; 2 Food Biotechnology Department, Instituto de la Grasa (C.S.I.C.), University Campus Pablo de Olavide, Building 46, Crta. de Utrera km 1, 41013 Seville, Spain

**Keywords:** Ascomycota, Biofilms, Genome plasticity, Methylotrophic yeast, Table olives

## Abstract

*Candida boidinii *is an Ascomycota yeast with important biotechnological applications. In this paper we present the genome sequencing and annotation of eight strains of this species isolated from human activities and wild environments. The produced assemblies revealed several strain specific features in terms of genomic GC content (ranging from 30.9 to 32.7%), genome size (comprised between 18,791,129 and 19,169,086 bp) and total number of protein coding genes (ranging from 5819 to 5998), with putative assignation to their general KOG functional categories. The obtained data underlined the presence of two different groups for this species. The results reported herein provide new insights into the plasticity of the genome of this yeast species and represent a starting point for further studies in view of its biotechnological applications.

## Introduction

*Candida boidinii* is a yeast belonging to *Ascomycota* phylum of the Kingdom Fungi, class *Saccharomycetes*, order *Saccharomycetales*, phylogenetically related to the *Ogataea* clade. This yeast species was first identified in Spain from a wash of tree bark by Ramirez [1], albeit the ecology of this microorganism is widespread and it has been isolated from diverse substrates related to human activity (wine fermentations, olive manufacturing, tepache, etc.) and natural environments (soil, seawater, sap fluxes of many sugar rich tree species, etc.) [2].

*C. boidinii* is a yeast species with a clear biotechnological potential. Indeed, this xylose-consuming and methylotrophic yeast proved to be suitable for the study of genes related with methanol degradation [3–5]. Moreover, this species is involved in olive processing, where it exhibits different multifunctional features such as lipase activity [6], biofilm formation on fruit epidermis [7, 8] and co-aggregation with LAB species such as *Lactobacillus pentosus* [9, 10].

Intraspecific biodiversity appears to be a distinctive feature of the *C. boidinii* species. Indeed, Lee and Komagata [11] compared the electrophoretic profiles of enzymes expressed in diverse strains of this species, revealing the presence of two distinct groups. Lin et al. [12] studied 19 *C. boidinii* strains isolated from diverse sources and also identified two divergent clusters both in terms of molecular (DNA base composition, electrophoretic karyotype, RFLP of RNA genes) and chemical (cellular fatty acid composition and ubiquinone system) features. The authors even highlighted a distinctive chromosomal banding pattern for each strain. Finally, statistics reported by the CBS-KNAW Fungal Biodiversity Centre show an average similarity between *C. boidinii* strains of 97.61% for 26S rDNA sequences (*n* = 38), and 98.06% for ITS sequences (*n* = 25) (http://www.cbs.knaw.nl/Collections/).

The biotechnological potential of *C. boidinii* *,* together with its underlined biodiversity, urge to obtain more information on the genome of this *Ascomycota* yeast. In facts, at the time of writing, the genome sequences of only two *C. boidinii* strains were available, namely GF002 (isolated from sugarcane bagasse, Bioproject PRJNA299882, [13]), and JCM9604 (isolated from tanning fluid, Bioproject PRJDB3623). In order to fill this lack of information, we hereafter report the genomic sequence and annotation of eight additional *C. boidinii* strains that were isolated from both human activities and wild environments.

## Organism information

### Classification and features

**Fig. 1:**
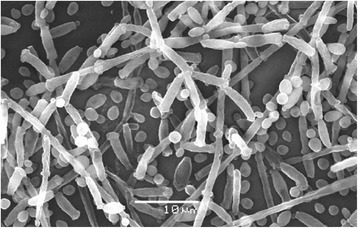
Scanning Electronic Microscopic image of the *C. boidinii* UNISS-Cb60 strain. Picture shows the morphology of single cells and pseudohyphae in YM broth medium after 7 days at 25 °C

**Fig. 2:**
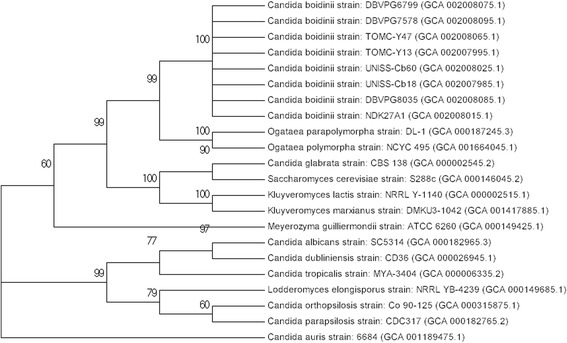
Phylogenetic position of the eight sequenced *C. boidinii* strains based on 18S rRNA sequences. Genbank accession numbers of the aligned sequences are indicated in brackets. *C. albicans* (strain MUCL29800) 18S rRNA (accession id X53497.1) was used as a query to retrieve the homologues sequences in the other presented species. Sequences were aligned using MUSCLE [37], and the phylogenetic tree was determined using the neighbour-joining algorithm with the Kimura 2-parameter distance model in MEGA (version 7) [38]. A gamma distribution (shape parameter = 1) was used for rate variation among sites. The optimal tree with the sum of branch lengths = 0.1734 is shown, and nodes that appeared in more than 50% of replicate trees in the bootstrap test (1000 replicates) are marked with their bootstrap support values

**Fig. 3:**
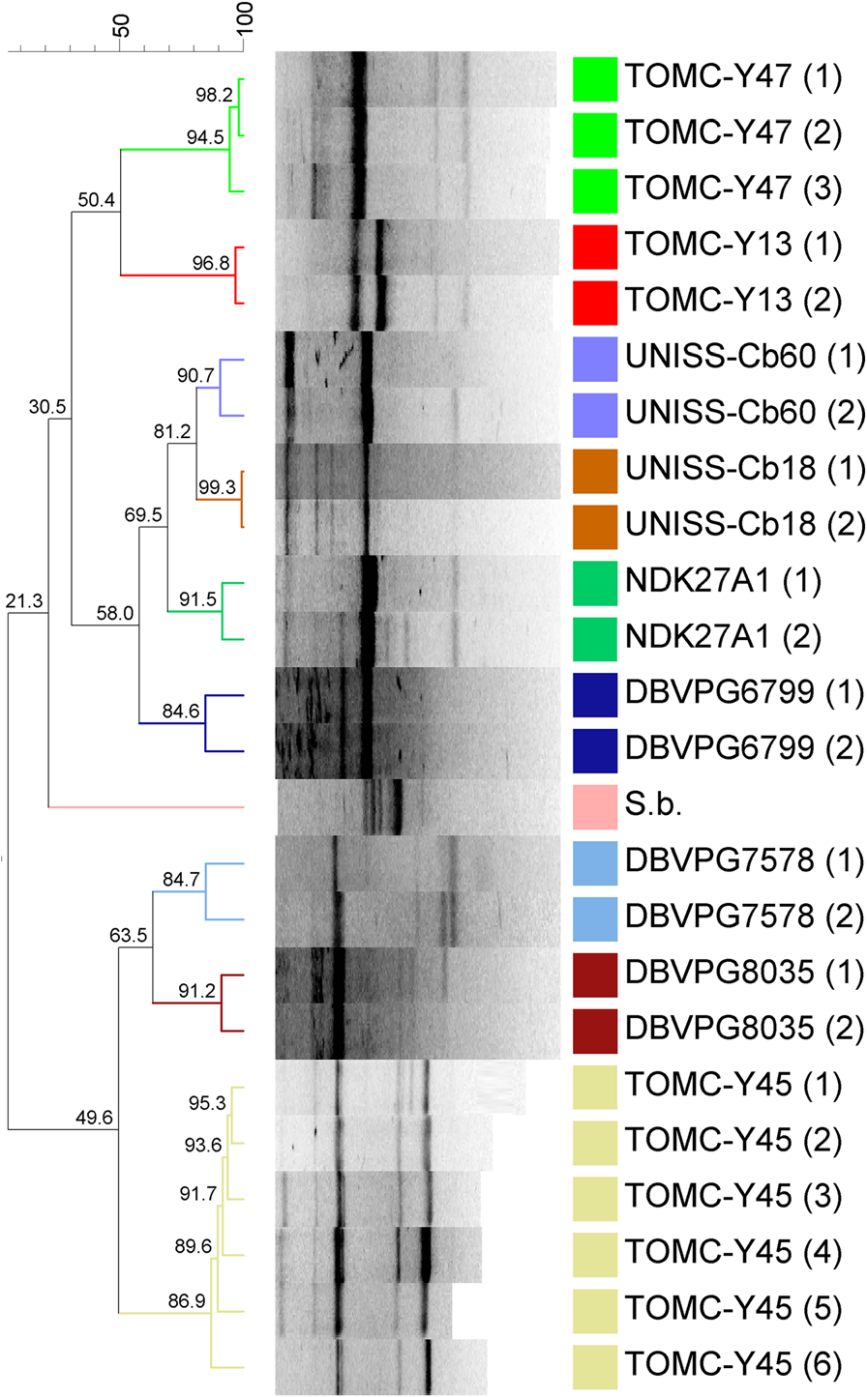
Dendrogram generated after bioinformatic analysis with Bionumerics 6.6 software package (Applied Maths, Kortrijk, Belgium) of the RAPD-PCR profiles obtained with M13 primer for the different strains of *C. boidinii.* Strains Sb (*Saccharomyces boulardii)* and TOMC-Y45 (*Wickerhamomyces anomalus*) were used as controls. Different profiles were also obtained for each *C. boidinii* strains to determine the reproducibility of the technique. Brackets specify the number of replicates for each strain

After previous studies on the ability of diverse yeast species to co-aggregate with diverse *Lactobacillus pentosus* strains [9] isolated from table olive fermentations, we selected eight strains of *C. boidinii* featuring different origins and degrees of co-aggregation. Strains UNISS-Cb18 and UNISS-Cb60 were obtained from the UNISS microbial collection (Università degli Studi di Sassari, Italy), TOMC-Y13 and TOMC-Y47 belong to the Table Olive Microorganisms Collection (Instituto de la Grasa-CSIC, Seville, Spain), DBVPG6799, DBVPG7578, and DBVPG8035 were obtained from the Industrial Yeast Collection (Università degli Studi di Perugia, Italy), and strain NDK27A1 was obtained from the Yeast Collection of the Dipartimento di Agraria (Università degli Studi di Naples, Italy). Tables 1, 2, 3, 4, 5, 6, 7 and 8 summarizes the classification, origin and main features of the studied organisms, whereas Fig. 1 shows, as an example, the morphology of one of the analysed strains (e.g. UNISS-Cb60) by scanning electron microscopy. Figure 2 shows the phylogenetic position of the selected *C. boidinii* isolates with respect to other yeast species, confirming its closely relationship with the *Ogataea* clade. The result presented here is originated by the alignment of the 18S rRNA sequences (Fig. 2); *C. albicans* (strain MUCL29800) 18S rRNA gene (accession id X53497.1), was used as a query to retrieve the homologues sequences within the other species assemblies (low coverage alignment prevented the inclusion of the published *C. boidinii* strain in the analysis). The observed phylogenetic closeness of the *C. boidinii* to the *Ogataea* clade was confirmed by the alignment of the D1/D2 domain of 26S rRNA gene (Additional file 1: Figure S1). Figure 3 shows the genotyping of these strains by RAPD-PCR analysis with M13 primers. All the strains were clearly grouped into different clusters for a cut-off value of 84.6% (the lowest reproducibility value was obtained between replicates for strain DBVPG6799).

**Table 1:** Classification and general features of the *Candida boidinii* strain UNISS-Cb18 according to the MIGS recommendations [39]

MIGS ID	Property	Term	Evidence code^a^
	Classification	Domain *Eukaryota*	
		Kingdom *Fungi*	TAS [40]
		Phylum *Ascomycota*	TAS [41]
		Class *Saccharomycetes*	TAS [42]
		Order *Saccharomycetales*	TAS [43]
		Family *Pichiaceae*	TAS [44]
		Genus *Candida (Tax ID: 1540042)*	TAS [45]
		Species *Candida boidinii*	TAS [1]
		Strain*: UNISS-Cb18*	
	Cell shape	*Long-ovoidal to cylindrical single, in pairs and chains. Pseudohyphae consisting of long branched chains of cells with verticals of ovoid blastoconidia*	TAS [2]
	Motility	*Non-motility*	TAS [2]
	Reproduction	*Asexual*	TAS [2]
	Temperature range	*15–37 °C*	NAS
	Optimum temperature	*25–30 °C*	TAS [2]
	pH range: optimum	*Not determined*	
	Carbon source	*multiple carbon sources*	TAS [2]
MIGS-6	Habitat	*Natural black table olive fermentation*	NAS
MIGS-6.3	Salinity	*Salt-tolerant*	IDA
MIGS-22	Oxygen requirement	*Aerobic, facultative anaerobic*	TAS [2]
MIGS-15	Biotic relationship	*free-living, biofilms*	TAS [2, 10]
MIGS-14	Pathogenicity	*Not reported*	NAS
MIGS-4	Geographic location	*Italy/Sardinia*	NAS
MIGS-5	Sample collection	*2003*	NAS
MIGS-4.1	Latitude	*Not determined*	
MIGS-4.2	Longitude	*Not determined*	
MIGS-4.4	Altitude	*Not determined*	

^a^Evidence codes – *IDA* Inferred from Direct Assay, *TAS* Traceable Author Statement (i.e., a direct report exists in the literature), *NAS* Non-traceable Author Statement (i.e., not directly observed for the living, isolated sample, but based on a generally accepted property for the species, or anecdotal evidence). These evidence codes are from the Gene Ontology project [46]

**Table 2:** Classification and general features of the *Candida boidinii* strain UNISS-Cb60 according to the MIGS recommendations [39]

MIGS ID	Property	Term	Evidence code^a^
	Classification	Domain *Eukaryota*	
		Kingdom *Fungi*	TAS [40]
		Phylum *Ascomycota*	TAS [41]
		Class *Saccharomycetes*	TAS [42]
		Order *Saccharomycetales*	TAS [43]
		Family *Pichiaceae*	TAS [44]
		Genus *Candida (Tax ID: 1540042)*	TAS [45]
		Species *Candida boidinii*	TAS [1]
		Strain*: UNISS-Cb60*	
	Cell shape	*Long-ovoidal to cylindrical single, in pairs and chains. Pseudohyphae consisting of long branched chains of cells with verticals of ovoid blastoconidia*	TAS [2]
	Motility	*Non-motility*	TAS [2]
	Reproduction	*Asexual*	TAS [2]
	Temperature range	*15–37 °C*	NAS
	Optimum temperature	*25–30 °C*	TAS [2]
	pH range: optimum	*Not determined*	
	Carbon source	*multiple carbon sources*	TAS [2]
MIGS-6	Habitat	*Natural black table olive fermentation*	NAS
MIGS-6.3	Salinity	*Salt-tolerant*	IDA
MIGS-22	Oxygen requirement	*Aerobic, facultative anaerobic*	TAS [2]
MIGS-15	Biotic relationship	*free-living, biofilms*	TAS [2, 10]
MIGS-14	Pathogenicity	*Not reported*	NAS
MIGS-4	Geographic location	*Italy/Sardinia*	NAS
MIGS-5	Sample collection	*2003*	NAS
MIGS-4.1	Latitude	*Not determined*	
MIGS-4.2	Longitude	*Not determined*	
MIGS-4.4	Altitude	*Not determined*	

^a^Evidence codes – *IDA* Inferred from Direct Assay, *TAS* Traceable Author Statement (i.e., a direct report exists in the literature), *NAS* Non-traceable Author Statement (i.e., not directly observed for the living, isolated sample, but based on a generally accepted property for the species, or anecdotal evidence). These evidence codes are from the Gene Ontology project [46]

**Table 3:** Classification and general features of the *Candida boidinii* strain TOMC-Y13 according to the MIGS recommendations [39]

MIGS ID	Property	Term	Evidence code^a^
	Classification	Domain *Eukaryota*	
		Kingdom *Fungi*	TAS [40]
		Phylum *Ascomycota*	TAS [41]
		Class *Saccharomycetes*	TAS [42]
		Order *Saccharomycetales*	TAS [43]
		Family *Pichiaceae*	TAS [44]
		Genus *Candida (Tax ID: 1540042)*	TAS [45]
		Species *Candida boidinii*	TAS [1]
		Strain*: TOMC-Y13*	
	Cell shape	*Long-ovoidal to cylindrical single, in pairs and chains. Pseudohyphae consisting of long branched chains of cells with verticals of ovoid blastoconidia*	TAS [2]
	Motility	*Non-motility*	TAS [2]
	Reproduction	*Asexual*	TAS [2]
	Temperature range	*15–37 °C*	NAS
	Optimum temperature	*25–30 °C*	TAS [2]
	pH range: optimum	*Not determined*	
	Carbon source	*multiple carbon sources*	TAS [2]
MIGS-6	Habitat	*Natural green table olive fermentation*	NAS
MIGS-6.3	Salinity	*Salt-tolerant*	IDA
MIGS-22	Oxygen requirement	*Aerobic, facultative anaerobic*	TAS [2]
MIGS-15	Biotic relationship	*free-living, biofilms*	TAS [2, 10]
MIGS-14	Pathogenicity	*Not reported*	NAS
MIGS-4	Geographic location	*Spain/Seville*	NAS
MIGS-5	Sample collection	*2011*	NAS
MIGS-4.1	Latitude	*Not determined*	
MIGS-4.2	Longitude	*Not determined*	
MIGS-4.4	Altitude	*Not determined*	

^a^Evidence codes – *IDA* Inferred from Direct Assay, *TAS* Traceable Author Statement (i.e., a direct report exists in the literature), *NAS* Non-traceable Author Statement (i.e., not directly observed for the living, isolated sample, but based on a generally accepted property for the species, or anecdotal evidence). These evidence codes are from the Gene Ontology project [46]

**Table 4:** Classification and general features of the *Candida boidinii* strain TOMC-Y47 according to the MIGS recommendations [39]

MIGS ID	Property	Term	Evidence code^a^
	Classification	Domain *Eukaryota*	
		Kingdom *Fungi*	TAS [40]
		Phylum *Ascomycota*	TAS [41]
		Class *Saccharomycetes*	TAS [42]
		Order *Saccharomycetales*	TAS [43]
		Family *Pichiaceae*	TAS [44]
		Genus *Candida (Tax ID: 1540042)*	TAS [45]
		Species *Candida boidinii*	TAS [1]
		Strain*: TOMC-Y47*	
	Cell shape	*Long-ovoidal to cylindrical single, in pairs and chains. Pseudohyphae consisting of long branched chains of cells with verticals of ovoid blastoconidia*	TAS [2]
	Motility	*Non-motility*	TAS [2]
	Reproduction	*Asexual*	TAS [2]
	Temperature range	*15–37 °C*	NAS
	Optimum temperature	*25–30 °C*	TAS [2]
	pH range: optimum	*Not determined*	
	Carbon source	*multiple carbon sources*	TAS [2]
MIGS-6	Habitat	*Directly brined table olive packaging*	NAS
MIGS-6.3	Salinity	*Salt-tolerant*	IDA
MIGS-22	Oxygen requirement	*Aerobic, facultative anaerobic*	TAS [2]
MIGS-15	Biotic relationship	*free-living, biofilms*	TAS [2, 10]
MIGS-14	Pathogenicity	*Not reported*	NAS
MIGS-4	Geographic location	*Spain/Málaga*	NAS
MIGS-5	Sample collection	*2014*	NAS
MIGS-4.1	Latitude	*Not determined*	
MIGS-4.2	Longitude	*Not determined*	
MIGS-4.4	Altitude	*Not determined*	

^a^Evidence codes – *IDA* Inferred from Direct Assay, *TAS* Traceable Author Statement (i.e., a direct report exists in the literature); *NAS* Non-traceable Author Statement (i.e., not directly observed for the living, isolated sample, but based on a generally accepted property for the species, or anecdotal evidence). These evidence codes are from the Gene Ontology project [46]

**Table 5:** Classification and general features of the *Candida boidinii* strain DBVPG6799 according to the MIGS recommendations [39]

MIGS ID	Property	Term	Evidence code^a^
	Classification	Domain *Eukaryota*	
		Kingdom *Fungi*	TAS [40]
		Phylum *Ascomycota*	TAS [41]
		Class *Saccharomycetes*	TAS [42]
		Order *Saccharomycetales*	TAS [43]
		Family *Pichiaceae*	TAS [44]
		Genus *Candida (Tax ID: 1540042)*	TAS [45]
		Species *Candida boidinii*	TAS [1]
		Strain*: DBVPG6799*	
	Cell shape	*Long-ovoidal to cylindrical single, in pairs and chains. Pseudohyphae consisting of long branched chains of cells with verticals of ovoid blastoconidia*	TAS [2]
	Motility	*Non-motility*	TAS [2]
	Reproduction	*Asexual*	TAS [2]
	Temperature range	*15–37 °C*	NAS
	Optimum temperature	*25–30 °C*	TAS [2]
	pH range: optimum	*Not determined*	
	Carbon source	*multiple carbon sources*	TAS [2]
MIGS-6	Habitat	*Cactus Opuntia sp.*	NAS
MIGS-6.3	Salinity	*Salt-tolerant*	IDA
MIGS-22	Oxygen requirement	*Aerobic, facultative anaerobic*	TAS [2]
MIGS-15	Biotic relationship	*free-living, biofilms*	TAS [2, 10]
MIGS-14	Pathogenicity	*Not reported*	NAS
MIGS-4	Geographic location	*Italy*	NAS
MIGS-5	Sample collection	*1992*	NAS
MIGS-4.1	Latitude	*Not determined*	
MIGS-4.2	Longitude	*Not determined*	
MIGS-4.4	Altitude	*Not determined*	

^a^Evidence codes – *IDA* Inferred from Direct Assay, *TAS* Traceable Author Statement (i.e., a direct report exists in the literature), *NAS* Non-traceable Author Statement (i.e., not directly observed for the living, isolated sample, but based on a generally accepted property for the species, or anecdotal evidence). These evidence codes are from the Gene Ontology project [46]

**Table 6:** Classification and general features of the *Candida boidinii* strain DBVPG7578 according to the MIGS recommendations [39]

MIGS ID	Property	Term	Evidence code^a^
	Classification	Domain *Eukaryota*	
		Kingdom *Fungi*	TAS [40]
		Phylum *Ascomycota*	TAS [41]
		Class *Saccharomycetes*	TAS [42]
		Order *Saccharomycetales*	TAS [43]
		Family *Pichiaceae*	TAS [44]
		Genus *Candida (Tax ID: 1540042)*	TAS [45]
		Species *Candida boidinii*	TAS [1]
		Strain*: DBVPG7578*	
	Cell shape	*Long-ovoidal to cylindrical single, in pairs and chains. Pseudohyphae consisting of long branched chains of cells with verticals of ovoid blastoconidia*	TAS [2]
	Motility	*Non-motility*	TAS [2]
	Reproduction	*Asexual*	TAS [2]
	Temperature range	*15–37 °C*	NAS
	Optimum temperature	*25–30 °C*	TAS [2]
	pH range: optimum	*Not determined*	
	Carbon source	*multiple carbon sources*	TAS [2]
MIGS-6	Habitat	*Soil*	NAS
MIGS-6.3	Salinity	*Salt-tolerant*	IDA
MIGS-22	Oxygen requirement	*Aerobic, facultative anaerobic*	TAS [2]
MIGS-15	Biotic relationship	*free-living, biofilms*	TAS [2, 10]
MIGS-14	Pathogenicity	*Not reported*	NAS
MIGS-4	Geographic location	*Russia*	NAS
MIGS-5	Sample collection	*1998*	NAS
MIGS-4.1	Latitude	*Not determined*	
MIGS-4.2	Longitude	*Not determined*	
MIGS-4.4	Altitude	*Not determined*	

^a^Evidence codes – *IDA* Inferred from Direct Assay, *TAS* Traceable Author Statement (i.e., a direct report exists in the literature); *NAS* Non-traceable Author Statement (i.e., not directly observed for the living, isolated sample, but based on a generally accepted property for the species, or anecdotal evidence). These evidence codes are from the Gene Ontology project [46]

**Table 7:** Classification and general features of the *Candida boidinii* strain DBVPG8035 according to the MIGS recommendations [39]

MIGS ID	Property	Term	Evidence code^a^
	Classification	Domain *Eukaryota*	
		Kingdom *Fungi*	TAS [40]
		Phylum *Ascomycota*	TAS [41]
		Class *Saccharomycetes*	TAS [42]
		Order *Saccharomycetales*	TAS [43]
		Family *Pichiaceae*	TAS [44]
		Genus *Candida (Tax ID: 1540042)*	TAS [45]
		Species *Candida boidinii*	TAS [1]
		Strain*: DBVPG8035*	
	Cell shape	*Long-ovoidal to cylindrical single, in pairs and chains. Pseudohyphae consisting of long branched chains of cells with verticals of ovoid blastoconidia*	TAS [2]
	Motility	*Non-motility*	TAS [2]
	Reproduction	*Asexual*	TAS [2]
	Temperature range	*15–37 °C*	NAS
	Optimum temperature	*25–30 °C*	TAS [2]
	pH range: optimum	*Not determined*	
	Carbon source	*multiple carbon sources*	TAS [2]
MIGS-6	Habitat	*Fresh water lake*	NAS
MIGS-6.3	Salinity	*Salt-tolerant*	IDA
MIGS-22	Oxygen requirement	*Aerobic, facultative anaerobic*	TAS [2]
MIGS-15	Biotic relationship	*free-living, biofilms*	TAS [2, 10]
MIGS-14	Pathogenicity	*Not reported*	NAS
MIGS-4	Geographic location	*Brazil*	NAS
MIGS-5	Sample collection	*2011*	NAS
MIGS-4.1	Latitude	*Not determined*	
MIGS-4.2	Longitude	*Not determined*	
MIGS-4.4	Altitude	*Not determined*	

^a^Evidence codes – *IDA* Inferred from Direct Assay, *TAS* Traceable Author Statement (i.e., a direct report exists in the literature); *NAS* Non-traceable Author Statement (i.e., not directly observed for the living, isolated sample, but based on a generally accepted property for the species, or anecdotal evidence). These evidence codes are from the Gene Ontology project [46]

**Table 8:** Classification and general features of the *Candida boidinii* strain NDK27A1 according to the MIGS recommendations [39]

MIGS ID	Property	Term	Evidence code^a^
	Classification	Domain *Eukaryota*	
		Kingdom *Fungi*	TAS [40]
		Phylum *Ascomycota*	TAS [41]
		Class *Saccharomycetes*	TAS [42]
		Order *Saccharomycetales*	TAS [43]
		Family *Pichiaceae*	TAS [44]
		Genus *Candida (Tax ID: 1540042)*	TAS [45]
		Species *Candida boidinii*	TAS [1]
		Strain*: NDK27A1*	
	Cell shape	*Long-ovoidal to cylindrical single, in pairs and chains. Pseudohyphae consisting of long branched chains of cells with verticals of ovoid blastoconidia*	TAS [2]
	Motility	*Non-motility*	TAS [2]
	Reproduction	*Asexual*	TAS [2]
	Temperature range	*15–37 °C*	NAS
	Optimum temperature	*25–30 °C*	TAS [2]
	pH range: optimum	*Not determined*	
	Carbon source	*multiple carbon sources*	TAS [2]
MIGS-6	Habitat	*Wine fermentation*	NAS
MIGS-6.3	Salinity	*Salt-tolerant*	IDA
MIGS-22	Oxygen requirement	*Aerobic, facultative anaerobic*	TAS [2]
MIGS-15	Biotic relationship	*free-living, biofilms*	TAS [2, 10]
MIGS-14	Pathogenicity	*Not reported*	NAS
MIGS-4	Geographic location	*Italy/Naples*	NAS
MIGS-5	Sample collection	*2015*	NAS
MIGS-4.1	Latitude	*Not determined*	
MIGS-4.2	Longitude	*Not determined*	
MIGS-4.4	Altitude	*Not determined*	

^a^Evidence codes – *IDA* Inferred from Direct Assay, *TAS* Traceable Author Statement (i.e., a direct report exists in the literature), *NAS* Non-traceable Author Statement (i.e., not directly observed for the living, isolated sample, but based on a generally accepted property for the species, or anecdotal evidence). These evidence codes are from the Gene Ontology project [46]

**Fig. 4:**
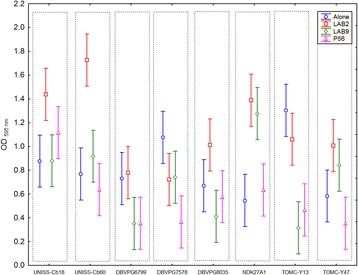
ANOVA analysis for the ability to form biofilms (OD_595nm_) of the eight *C. bodinii* strains studied in this work. The plot shows the ability to form biofilm of the analysed strains alone or in combination with *L. pentosus* TOMC-LAB2 (LAB2), *L. plantarum* TOMC-LAB9 (LAB9), and *P. pentosaceus* TOMC-P56 (P56). Error bars were obtained from six replicate measurements for each treatment

The specific ability of the eight *C. boidinii* strains to form biofilm alone or in combination with three LAB strains isolated from table olives (*L. pentosus* TOMC-LAB2, *Lactobacillus plantarum* TOMC-LAB9, and *Pediococcus pentosaceus* TOMC-P56) was quantified by crystal violet staining. Briefly, 96-well microtiter plates were inoculated with 100 μL of overnight culture of each *C. boidinii* strain, alone or in combination with 100 μL of the mentioned LAB. After 48 h incubation at 28 °C, liquid was removed from wells and washed twice with sterile saline solution (0.9%). Subsequently, a crystal violet solution (0.8% *w*/*v*) was added to each well. Plates were incubated at room temperature for 30 min and then washed twice with sterile distilled water. Finally, an ethanol-acetone mixture (80:20, *v*/v) was added in order to extract crystal violet bound to biofilm. After 30 min incubation at room temperature, the OD at 595 nm was determined with a spectrophotometer model Spectrostar Nano (BMG Labtech, Ortemberg Germany). Multifactorial ANOVA was used to compare OD values obtained for the different strains. Results are shown in Fig. 4. As clearly deduced, different ability to form biofilms was exhibited among strains. In mono-culture, the lowest value was obtained for strain NDK27A1 (OD 0.5), which was statistically different compared to the strain with the highest value (TOMC-Y13, OD 1.3). Moreover, for many of the strains, biofilm production was statistically higher in mixed culture in presence of the *L. pentosus* species, which was especially evident for strains UNISS-Cb18, UNISS-Cb60, and NDK27A1. This fact did not occur for the other LAB species. Only for strain NDK27A1, the presence of *L. plantarum* also produced a considerable increase in its ability to form biofilm.

## Genome sequencing information

### Genome project history

Formation of mixed biofilms between yeasts and LAB on the surface of olives during the fermentation process is a widely observed phenomenon [8]. This phenotype is determined by the expression of multiple genes of both the bacteria and the yeast. In this regard, *C. boidinii* has been described as a yeast with high ability to form mixed biofilms [10] and, for this reason, several strains were sequenced aiming to investigate in further studies the genetic bases of the observed peculiar behaviour. The genome project was deposited under the accession number PRJNA359406. Tables 9 and 10 shows a summary of this genome project, which encompassed for a total of eight microorganisms.

**Table 9:** Project information for the *C. boidinii* strains UNISS-Cb18, UNISS-Cb60, TOMC-Y13, and TOMC-Y47

MIGS ID	Property	UNISS-Cb18	UNISS-Cb60	TOMC-Y13	TOMC-Y47
MIGS 31	Finishing quality	High-quality draft			
MIGS-28	Libraries used	Nextera XT paired end Library			
MIGS 29	Sequencing platforms	Illumina MiSeq			
MIGS 31.2	Fold coverage	93×	80×	64×	68×
MIGS 30	Assemblers	SPAdes v. 3.8.2			
MIGS 32	Gene calling method	Augustus v. 2.5.5			
	Locus Tag	–			
	Genbank ID	MSRX00000000	MSRY00000000	MSRZ00000000	MSSA00000000
	GenBank Date of Release	03/01/17			
	GOLD ID	–			
	BIOPROJECT	PRJNA359406			
MIGS 13	Source Material Identifier	UNISS-Cb18	UNISS-Cb60	TOMC-Y13	TOMC-Y47
	Project relevance	Industrial

**Table 10:** Project information for the *C. boidinii* strains DBVPG6799, DBVPG7578, DBVPG8035, and NDK27A1

MIGS ID	Property	DBVPG6799	DBVPG7578	DBVPG8035	NDK27A1
MIGS 31	Finishing quality	High-quality draft			
MIGS-28	Libraries used	Nextera XT paired end Library			
MIGS 29	Sequencing platforms	Illumina MiSeq			
MIGS 31.2	Fold coverage	74×	72×	91×	113×
MIGS 30	Assemblers	SPAdes v. 3.8.2			
MIGS 32	Gene calling method	Augustus v. 2.5.5			
	Locus Tag	–			
	Genbank ID	MSSB00000000	MSSC00000000	MSSD00000000	MSSE00000000
	GenBank Date of Release	03/01/17			
	GOLD ID	–			
	BIOPROJECT	PRJNA359406			
MIGS 13	Source Material Identifier	DBVPG6799	DBVPG7578	DBVPG8035	NDK27A1
	Project relevance	Industrial			

### Growth conditions and genomic DNA preparation

DNA extraction of the *C. boidinii* strains was performed according to Borelli et al. [13] with slight modifications. First, yeasts strains were grown in YM broth medium (Difco, Becton and Dickinson Company, Sparks, MD, USA) at 28 °C, centrifuged, and then the cells washed with 1 mL of sterile MilliQ ultrapure water. Washed cells were collected at 15,000 rpm for 10 min at 4 °C. After removal of the supernatant, 200 μL of lysis buffer (2% Triton-X-100 [v/v], 1% SDS [v/v], 100 mM NaCl, 10 mM TrisHCl [pH 8.0], 1 mM EDTA [pH 8.0]), 0.3 g of glass beads, and 200 μL of phenol:chloroform:isoamyl-alcohol (25:24:1, v/v) were added to the pellets. After vortexing for 2 min, 200 μL of TE buffer (10 mM Tris-HCl, 1 mM EDTA [pH 8.0]) were added. It was followed by centrifugation at 15,000 rpm for 10 min at 4 °C. The supernatants were then transferred into new tubes, where 3 μL of RNase (10 μg/mL) (Sigma-Aldrich) were added and the mixture was incubated at 37 °C for 30 min. After incubation, total DNA was precipitated with 18 μL of sodium acetate (3 M, pH 5.3) and 400 μL of cold ethanol 100%. After centrifugation (15,000 rpm, 15 min, 4 °C) the supernatants were discarded and DNA pellets were washed with ethanol 70%. DNA pellets were suspended in 50 μL of TE buffer. The concentration and quality of extracted DNA were evaluated using a Spectrostar NANO spectrophotometer (BMG LABTECH. Ortemberg, Germany) at 260_nm_ and by agarose gel electrophoresis (data not shown).

### Genome sequencing and assembly

Whole genome sequencing was performed at the FISABIO Sequencing and Bioinformatics services (Valencia , Spain) using Illumina Miseq technology. DNA libraries were generated following the Nextera XT Illumina protocol (Nextera XT Library Prep kit [FC-131-1024]). Purified yeast genomic DNA (0.2 ng μl^−1^) was used to initiate the protocol. The libraries were sequenced using a 2 × 300 bp paired-end run (MiSeq Reagent kit v3 [MS-102-3001]) on a MiSeq Sequencer according to manufacturer’s instructions. The produced 51,248,190 bp reads for the eight *C. boidinii* strains (see Table S1 in Additional file 2 for more details) were quality-filtered using prinseq-lite program [14] applying the following parameters: min_length: 50, trim_qual_right: 30, trim_qual_type: mean, trim_qual_window: 20). Then, R1 and R2 from Illumina sequencing where joined using fastq-join from ea-tools suite (https://expressionanalysis.github.io/ea-utils/) applying the following default parameters: maximum percent difference: 8, minimum overlap: 6. The resulting datasets were used to assemble all the *C. boidinii* strains’ genomes by using the software SPAdes [15]. Scaffolds that proved to be shorter than 500 bp were removed from the final assembly.

### Genome annotation

The obtained genomes were annotated using the tool Augustus [16] that was trained with transcripts from *Candida tropicalis*. Such a species was chosen among others (e.g. *Candida albicans* and *Candida guilliermondii* from the built-in Augustus training sets and *Candida glabrata* from an ad hoc training set derived from the gene models available at the NCBI genome database) based on the number of predicted genes showing high homology (blastp search, *e*-value < 0.0001, Additional file 3: Table S2) with a dataset of proteins annotated in several yeasts species (e.g. *C. dublinensis, C. albicans, C. glabrata, C. guilliermondii, C. lusitaniae, C. orthopsilosis, C. parapsilosis, C. tropicalis, D. hansenii, D. kurascia, L. elongisporus, P. tannophilus, P. membranifaciens*). Reliability of prediction was confirmed by a remarkable concordance of the predicted exonic ranges among different training sets (e.g. 98% of the exons predicted using *C. tropicalis* as the training set proved to be consistent with exons predicted with *C. glabrata* as training set). Transfer RNA and ribosomal RNA were predicted by using the software tRNAscan [17] and RNAmmer [18] respectively. The tool Blast2GO [19] was used to assign a putative function to the predicted transcripts either in terms of molecular function, cellular component or biological process. The presence of Pfam domains [20] was investigated by the use of the Batch Web CD-Search Tool from NCBI [21], whereas KOG functional categorization was achieved using the WebMGA web server [22]. Finally, CRISPRFinder [23], SignalP 4.1 server [24] and TMHMM server [25] were used to investigate the presence of CRISPR repeats, signal peptides and transmembrane domains, respectively, within the predicted genes. RepeatModeler [26] was used to investigate the presence of transposable elements in the eight investigated *C. boidinii* species; the retrieved sequences were merged with the Repbase fungi transposable elements dataset [27] and the resulting library was used to perform a full analysis of the *C. boidinii* strains repetitive regions by using the RepeatMasker tool [28].

## Genome properties

Assembly of the eight *C. boidinii* strains’ draft genomes produced between 235 (UNISS-Cb60) and 860 (TOMC-Y13) scaffolds. The genomes’ lengths were approximately 18,800,000 bp for strains UNISS-Cb18, UNISS-Cb60, DBVPG6799, and NDK27A1 and around 19,100,000 for all the remaining species (Table 11). Strains UNISS-Cb18, UNISS-Cb60, and NDK27A1 proved to have the highest genomic GC content (32.66, 32.65, and 32.68% respectively) compared to the other sequenced species (~ 31%). The number of predicted protein coding sequences varied between 5819 (UNISS-Cb18) and 5998 (TOMC-Y13). The software Blast2GO allowed identify valid ontology terms for a percentage of genes ranging from 65.67 to 67.07. Further properties of the predicted genes are reported in Table 11, whereas functional classification into KOG categories is reported in Tables 12 and 13. Finally data relative to the transposable elements, simple repeats and low complexity regions are reported in Additional file 4: Table S3.

**Table 11:** Genome statistics

Attribute	UNISS-Cb18		UNISS-Cb60		TOMC-Y13	
	Value	% of Total	Value	% of Total	Value	% of Total
Genome size (bp)	18,791,961	100	18,794,311	100	18,987,836	100
DNA coding (bp)	9,828,418	52.3	9,838,412	52.35	9,664,304	50.9
DNA G + C (bp)	6,137,862	32.66	6,136,696	32.65	5,889,163	31.02
DNA scaffolds	279	100	235	100	860	100
Total genes	6112	100	6171	100	6343	100
Protein coding genes	5819	95.21	5827	94.43	5998	95.21
RNA genes	293	4.79	344	5.57	345	4.79
Pseudo genes	–	–	–	–	–	–
Genes in internal clusters	–	–	–	–	–	–
Genes with function prediction	3898	66.99	3908	67.07	3939	65.67
Genes assigned to COGs	4988	81.61	4991	80.88	5113	80.61
Genes with Pfam domains	4802	78.57	4802	77.82	4783	75.41
Genes with signal peptides	226	3.7	222	3.6	259	4.08
Genes with transm. helices	1094	17.9	1097	17.78	1041	16.41
CRISPR repeats	1	0.02	1	0.02	0	0
	TOMC-Y47		DBVPG6799		DBVPG7578	
	Value	% of Total	Value	% of Total	Value	% of Total
Genome size (bp)	19,120,811	100	18,807,174	100	19,169,086	100
DNA coding (bp)	9,775,915	51.13	9,805,165	52.14	9,784,744	51.04
DNA G + C (bp)	5,915,475	30.94	6,150,837	32.7	5,934,349	30.96
DNA scaffolds	597	100	431	100	628	100
Total genes	6327	100	6169	100	6301	100
Protein coding genes	5932	95.21	5888	95.21	5963	95.21
RNA genes	395	4.79	281	4.79	338	4.79
Pseudo genes	–	–	–	–	–	–
Genes in internal clusters	–	–	–	–	–	–
Genes with function prediction	3927	66.2	3889	66.05	3939	66.06
Genes assigned to COGs	5120	80.92	4988	80.86	5136	81.51
Genes with Pfam domains	4803	75.91	4804	77.87	4818	76.46
Genes with signal peptides	259	4.09	226	3.66	262	4.16
Genes with transm. helices	1114	17.61	1095	17.75	1127	17.89
CRISPR repeats	3	0.05	3	0.05	9	0.14
	DBVPG8035		NDK27A1			
	Value	% of Total	Value	% of Total		
Genome size (bp)	19,138,300	100	18,791,129	100		
DNA coding (bp)	9,827,091	51.35	9,871,244	52.53		
DNA G + C (bp)	5,914,797	30.91	6,140,718	32.68		
DNA scaffolds	557	100	272	100		
Total genes	6253	100	6132	100		
Protein coding genes	5922	95.21	5835	95.21		
RNA genes	331	4.79	297	4.79		
Pseudo genes	–	–	–	–		
Genes in internal clusters	–	–	–	–		
Genes with function prediction	3893	65.74	3907	66.96		
Genes assigned to COGs	5108	81.69	4985	81.29		
Genes with Pfam domains	4804	76.83	4820	78.6		
Genes with signal peptides	256	4.09	226	3.69		
Genes with transmem. helices	1122	17.94	1109	18.09		
CRISPR repeats	2	0.03	3	0.05

**Table 12:** Number of genes associated with general KOG functional categories for the *C. boidinii* strains UNISS-Cb18, UNISS-Cb60, TOMC-Y13, and TOMC-Y47

Code	UNISS-Cb18		UNISS-Cb60		TOMC-Y13		TOMC-Y47		Description
	Value	%age	Value	%age	Value	%age	Value	%age	
J	387	6.33	384	6.22	393	6.2	397	6.27	Translation, ribosomal structure and biogenesis
A	271	4.43	267	4.33	267	4.21	273	4.31	RNA processing and modification
K	654	10.7	657	10.65	678	10.69	683	10.8	Transcription
L	196	3.21	196	3.18	208	3.28	206	3.26	Replication, recombination and repair
B	103	1.69	106	1.72	114	1.8	115	1.82	Chromatin structure and dynamics
D	280	4.58	281	4.55	309	4.87	312	4.93	Cell cycle control, cell division, chromosome partitioning
Y	40	0.65	39	0.63	43	0.68	42	0.66	Nuclear structure
V	36	0.59	36	0.58	33	0.52	34	0.54	Defence mechanisms
T	384	6.28	382	6.19	374	5.9	376	5.94	Signal transduction mechanisms
M	57	0.93	58	0.94	66	1.04	62	0.98	Cell wall/membrane/envelope biogenesis
N	3	0.05	3	0.05	2	0.03	2	0.03	Cell motility
Z	166	2.72	163	2.64	169	2.66	170	2.69	Cytoskeleton
W	11	0.18	9	0.15	10	0.16	10	0.16	Extracellular structures
U	366	5.99	365	5.91	370	5.83	369	5.83	Intracellular trafficking, secretion, and vesicular transport
O	461	7.54	462	7.49	472	7.44	468	7.4	Post-translational modification, protein turnover, chaperones
C	232	3.8	232	3.76	236	3.72	240	3.79	Energy production and conversion
G	184	3.01	184	2.98	187	2.95	187	2.96	Carbohydrate transport and metabolism
E	248	4.06	252	4.08	252	3.97	253	4	Amino acid transport and metabolism
F	71	1.16	71	1.15	74	1.17	74	1.17	Nucleotide transport and metabolism
H	89	1.46	90	1.46	92	1.45	91	1.44	Coenzyme transport and metabolism
I	181	2.96	180	2.92	181	2.85	181	2.86	Lipid transport and metabolism
P	126	2.06	128	2.07	137	2.16	142	2.24	Inorganic ion transport and metabolism
Q	92	1.51	92	1.49	110	1.73	108	1.71	Secondary metabolites biosynthesis, transport and catabolism
R	643	10.52	645	10.45	643	10.14	646	10.21	General function prediction only
S	299	4.89	300	4.86	298	4.7	295	4.66	Function unknown
X	0	0	0	0	0	0	0	0	Multiple functions
–	0	0	0	0	0	0	0	0	Not in KOGs

**Table 13:** Number of genes associated with general KOG functional categories for the *C. boidinii* strains DBVPG6799, DBVPG7578, DBVPG8035, and NDK27A1

Code	DBVPG6799		DBVPG7578		DBVPG8035		NDK27A1		Description
	Value	%age	Value	%age	Value	%age	Value	%age	
J	379	6.14	393	6.24	385	6.16	392	6.39	Translation, ribosomal structure and biogenesis
A	281	4.56	272	4.32	269	4.3	272	4.44	RNA processing and modification
K	663	10.75	689	10.93	693	11.08	654	10.67	Transcription
L	197	3.19	205	3.25	202	3.23	197	3.21	Replication, recombination and repair
B	105	1.7	110	1.75	111	1.78	106	1.73	Chromatin structure and dynamics
D	293	4.75	303	4.81	296	4.73	292	4.76	Cell cycle control, cell division, chromosome partitioning
Y	39	0.63	42	0.67	38	0.61	44	0.72	Nuclear structure
V	32	0.52	35	0.56	35	0.56	35	0.57	Defence mechanisms
T	388	6.29	387	6.14	383	6.13	374	6.1	Signal transduction mechanisms
M	53	0.86	66	1.05	69	1.1	56	0.91	Cell wall/membrane/envelope biogenesis
N	3	0.05	2	0.03	2	0.03	3	0.05	Cell motility
Z	172	2.79	169	2.68	171	2.73	165	2.69	Cytoskeleton
W	11	0.18	12	0.19	9	0.14	9	0.15	Extracellular structures
U	366	5.93	367	5.82	364	5.82	366	5.97	Intracellular trafficking, secretion, and vesicular transport
O	465	7.54	477	7.57	482	7.71	458	7.47	Post-translational modification, protein turnover, chaperones
C	233	3.78	239	3.79	237	3.79	233	3.8	Energy production and conversion
G	188	3.05	187	2.97	185	2.96	183	2.98	Carbohydrate transport and metabolism
E	246	3.99	251	3.98	253	4.05	253	4.13	Amino acid transport and metabolism
F	70	1.13	75	1.19	74	1.18	71	1.16	Nucleotide transport and metabolism
H	89	1.44	92	1.46	94	1.5	92	1.5	Coenzyme transport and metabolism
I	179	2.9	180	2.86	180	2.88	180	2.94	Lipid transport and metabolism
P	127	2.06	139	2.21	140	2.24	126	2.05	Inorganic ion transport and metabolism
Q	92	1.49	112	1.78	102	1.63	91	1.48	Secondary metabolites biosynthesis, transport and catabolism
R	640	10.37	652	10.35	652	10.43	638	10.4	General function prediction only
S	289	4.68	297	4.71	293	4.69	298	4.86	Function unknown
X	0	0	0	0	0	0	0	0	Multiple functions
–	0	0	0	0	0	0	0	0	Not in KOGs

## Insights from the genome sequence

Sequencing data were used to compare the reported strains to the published genome of *C. boidinii* (strain GF002) [13]. The reads of each experiment were aligned to the reference genome by using the software bwa [29] with default parameters (edit distance = 4%). The obtained results highlighted the presence of two distinct groups. Indeed, while UNISS-Cb18, UNISS-Cb60, DBVPG6799 and NDK27A1 (hereafter referred to as group A) proved to share only 9% with the reference DNA sequence (with such a percentage increasing to around 50% when the most permissive aligner bwa mem was used), the remaining strains (TOMC-Y13, TOMC-Y47, DBVPG7578, and DBVPG8035, hereafter referred to as group B) proved to cover around 97% with the GF002 genome. Notably, these two groups also significantly differ in their GC content (*p* < 0.0001) and genome length (*p* < 0.001). Although the phylogenetic tree (Fig. 2) and the high level of D1D2 26S ribosomal sequence conservation within as well as between the two groups (Additional file 5: Table S4) show a clear strong phylogenetic relationship among the presented strains, the observed genetic diversity is not surprising. A marked GC content variability and the identification of two distinct groups (based on the chemo-variability derived from the electrophoretic patterns of several enzymes) was previously reported for this species [12].

### Extended insights

**Fig. 5:**
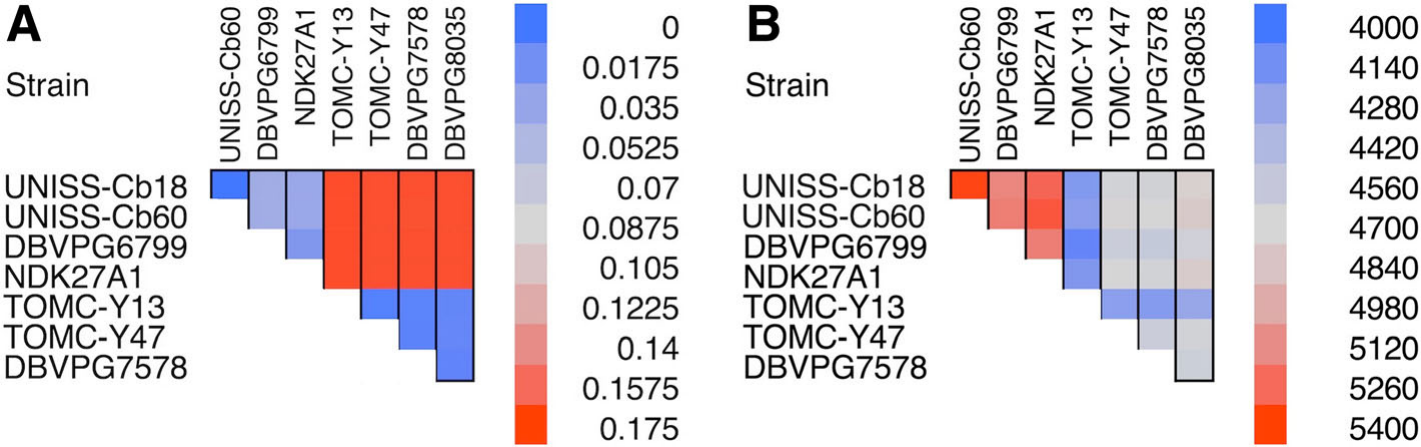
Heatmap describing the genomic diversity of the eight analysed *C. boidinii* strains. **a** Distance matrix calculated by considering the frequency of all possibile 25-mer sequences within the assembled genomes. **b** Number of collinear genes between the analysed strains

The emergence of two apparently distinct groups for the reported *C. boidinii* strains was further investigated by analysing their genetic diversity in terms of both nucleotide divergence and chromosomal structural variability. In this regard, we first computed the frequency of all possible k-mers (DNA substrings of a specific size k = 25) that are included in each of the assembled genomes by using the pipeline FFP (v. 3.19, [30]). Such an approach has been used to investigate the signature of genetic similarity by directly comparing several genomes even in the absence of a well characterized model organism. The obtained frequencies were used to compute a distance matrix (Fig. 5a) that clearly confirmed the strong similarity between strains belonging to the same group. We speculate that the observed compositional diversity can be due to different factors such as the strength of the mutational pressure [31], the effect of selection [32] or the incidence of the GC biased gene conversion [33]. In this regard, the occurrence of complex structural rearrangements can not be excluded either. For this reason, we used the OrthoMCL pipeline (with default parameters, [34]) to find the orthologues genes of the presented strains and studied their collinearity by using the tool MCscanX [35]. A low sinteny level generally underlie the occurrence of complex structural variation events such as genomic rearrangements or horizontal gene transfer [36]. The analysis involved a total of 47,184 genes and revealed that 88.2% of these were in a collinear group: however a large variability emerged when the collinear group were analysed for each pairs of species (Fig. 5b). The lowest number of collinear genes arose when strains belonging to different groups were compared. Notably, a very high number of genes proved to be collinear when analysing strains belonging to group A with such a trend being less marked for strains within group B and with strain TOMC-Y13 featuring, in general, the smallest values. As reported in Table 14, the sinteny analysis revealed several parameters discriminating the two groups such the number of dispersed genes (e.g. transcripts that are not collinear with any of the orthologues genes, A < B, *p* < 0.01), the occurrence of tandem duplications (A < B, *p* < 0.001) and the number of proximal genes (e.g. transcripts that are duplicated within the analysed species at a distance comprised between 2 and 20 genes, A > B, *p* < 0.001). The analysis of repetitive regions further confirmed such a discrimination (Additional file 4: Table S3) with group A featuring a higher number of LINE (*p* < 0.05), LTR (*p* < 0.001) but a lower number of simple repeats (*p* < 0.0001) and low complexity sequences (*p* < 0.0001). Taken together these results suggest an evident impact of complex structural variations in shaping the genome of the *C. boidini* with such a phenomenon conferring specific genomic structure to strains with diverse evolutionary histories.

**Table 14:** MCscanX classification of the genes for the eight *C. boidinii* strains

Strains	Group	Singletons	Dispersed	Proximal	Tandem
NDK27A1	A	1	210	48	128
DBVP6799		8	355	45	135
UNISS-Cb18		3	180	50	121
UNISS-Cb60		3	147	52	124
TOMC-Y13	B	6	1164	32	192
TOMC-Y47		5	658	31	177
DBVP7578		6	713	31	176
DBVP8035		11	557	24	179

## Conclusions

In this study, we have sequenced and characterized the genome of eight *C. boidinii* strains isolated from diverse origins and featuring peculiar co-aggregation behaviour. The analysed species featured a high variability in terms of nucleotide compositional patterns and genomic structure, possibily reflecting their specific evolutionary history. This result underline the need to deeply investigate the phylogenesis of the *C. boidinii* species by comparing the reported genomes to those of related species in terms of orthologues protein evolution or transcripts collinearity. The occurrence of both the strain specific duplicated genes and the singletons (e.g. genes with no orthologues in other strains) will need to be further investigated in order to study their involvement in the highlighted morphological differences. We strongly believe that generated data will boost future studies aiming the exploration of both the biotechnological potential and the genome plasticity of this *Ascomycota* yeast.

## Additional files

Additional file 1: Figure S1. Phylogenetic position of the eight sequenced *C. boidinii* strains based on D1/D2 domain of 26S rRNA sequences. Genbank assembly accession numbers of the aligned sequences are indicated in brackets. *C. boidinii* (strain SA18S03) D1/D2 domain (accession id EF460654.1) was used as a query to retrieve the homologues sequences in the other presented species. Low coverage alignment prevented the inclusion of the published *C. boidinii* strain in the analysis. Sequences were aligned using MUSCLE [37], and the phylogenetic tree was determined using the neighbour-joining algorithm with the Kimura 2-parameter distance model in MEGA (version 7) [38]. A gamma distribution (shape parameter = 1) was used for rate variation among sites. The optimal tree with the sum of branch lengths = 1.5319 is shown, and nodes that appeared in more than 50% of replicate trees in the bootstrap test (1000 replicates) are marked with their bootstrap support values. (TIFF 1387 kb)

Additional file 2: Table S1. Number of reads generated upon sequencing of eight *C. boidinii* strains. (DOCX 15 kb)

Additional file 3: Table S2. Number of predicted genes showing high homology (*e*-value < 0.0001) with gene models predicted in several Candida related species. The data refers to the analysis of strain Cb18 with four different Augustus training sets. (DOCX 14 kb)

Additional file 4: Table S3. Number of genomic bases included in transposable elements, simple repeats and low complexity regions of eight *C. boidinii* strains. (DOCX 14 kb)

Additional file 5: Table S4. Alignment statistics for the Blast search of two D1D2 ribosomal portions (isolated and sequenced from one high GC and one low GC content strain) in the eight *C. boidinii* strains. (DOCX 15 kb)

## Abbreviations

LAB: Lactic acid bacteria
OD: Optical density

## References

[1] Ramírez C. Estudio sobre nuevas especies de levaduras aisladas de diferentes sustratos. Microbiol Española. 1953;6:249–53.

[2] Kurtzman C, Fell JW, Boekhout T. The yeasts. Amsterdam: Elsevier; 2011.

[3] Vongsuvanlert V, Tani Y (1988). Purification and characterization of xylose isomerase of a methanol yeast, Candida boidinii, which is involved in sorbitol production from glucose. Agric Biol Chem.

[4] Grembecka M (2015). Sugar alcohols—their role in the modern world of sweeteners: a review. Eur Food Res Technol.

[5] Oda S, Yurimoto H, Nitta N, Sasano Y, Sakai Y (2015). Molecular characterization of hap complex components responsible for methanol-inducible gene expression in the methylotrophic yeast Candida boidinii. Eukaryot Cell.

[6] Rodríguez-Gómez F, Arroyo-López FN, López-López A, Bautista-Gallego J, Garrido-Fernández A (2010). Lipolytic activity of the yeast species associated with the fermentation/storage phase of ripe olive processing. Food Microbiol.

[7] Domínguez-Manzano J, León-Romero Á, Olmo-Ruiz C, Bautista-Gallego J, Arroyo-López FN, Garrido-Fernández A (2012). Biofilm formation on abiotic and biotic surfaces during Spanish style green table olive fermentation. Int J Food Microbiol.

[8] Arroyo-López FN, Bautista-Gallego J, Domínguez-Manzano J, Romero-Gil V, Rodríguez-Gómez F, García-García P (2012). Formation of lactic acid bacteria–yeasts communities on the olive surface during Spanish-style Manzanilla fermentations. Food Microbiol.

[9] Zanoni P, Farrow JAE, Phillips BA, Collins MD (1987). Lactobacillus pentosus, (Fred, Peterson and Anderson) sp. nov., nom. rev. Int J Syst Bacteriol.

[10] León-Romero Á, Domínguez-Manzano J, Garrido-Fernández A, Arroyo-López FN, Jiménez-Díaz R (2015). Formation of in vitro mixed-species biofilms by lactobacillus pentosus and yeasts isolated from Spanish-style green table olive fermentations. Appl Environ Microbiol.

[11] Lee J-D, Komagata K (1983). Further taxonomic study of methanol-assimilating yeasts with special references to electrophoretic comparison of enzymes. J Gen Appl Microbiol.

[12] Lin YH, Lee FL, Hsu WH (1996). Molecular and chemical taxonomic differentiation of Candida Boidinii Ramirez strains. Int J Syst Bacteriol.

[13] Borelli G, José J, Teixeira PJPL, dos Santos LV, Pereira GAG (2016). De novo assembly of Candida sojae and Candida boidinii genomes, unexplored Xylose-consuming yeasts with potential for renewable biochemical production. Genome Announc.

[14] Schmieder R, Edwards R (2011). Quality control and preprocessing of metagenomic datasets. Bioinformatics.

[15] Bankevich A, Nurk S, Antipov D, Gurevich AA, Dvorkin M, Kulikov AS (2012). SPAdes: a new genome assembly algorithm and its applications to single-cell sequencing. J Comput Biol.

[16] Keller O, Kollmar M, Stanke M, Waack S (2011). A novel hybrid gene prediction method employing protein multiple sequence alignments. Bioinformatics.

[17] Lowe TM, Eddy SR (1997). tRNAscan-SE: a program for improved detection of transfer RNA genes in genomic sequence. Nucleic Acids Res.

[18] Lagesen K, Hallin P, Rodland EA, Staerfeldt HH, Rognes T, Ussery DW (2007). RNAmmer: consistent and rapid annotation of ribosomal RNA genes. Nucleic Acids Res.

[19] Conesa A, Götz S, García-Gómez JM, Terol J, Talón M, Robles M (2005). Blast2GO: a universal tool for annotation, visualization and analysis in functional genomics research. Bioinformatics.

[20] Finn RD, Coggill P, Eberhardt RY, Eddy SR, Mistry J, Mitchell AL (2016). The Pfam protein families database: towards a more sustainable future. Nucleic Acids Res.

[21] Marchler-Bauer A (2004). CDD: a Conserved Domain Database for protein classification. Nucleic Acids Res.

[22] Wu S, Zhu Z, Fu L, Niu B, Li W (2011). WebMGA: a customizable web server for fast metagenomic sequence analysis. BMC Genomics.

[23] Grissa I, Vergnaud G, Pourcel C (2007). CRISPRFinder: a web tool to identify clustered regularly interspaced short palindromic repeats. Nucleic Acids Res.

[24] Petersen TN, Brunak S, Heijne v G, Nielsen H (2011). SignalP 4.0: discriminating signal peptides from transmembrane regions. Nat Methods.

[25] Krogh A, Larsson B, Heijne v G, Sonnhammer EL (2001). Predicting transmembrane protein topology with a hidden Markov model: application to complete genomes. J Mol Biol.

[26] Smit AFA, Hubley R. RepeatModeler Open.1–0. 2008–2015. http://www.repeatmasker.org. Accessed 24 Nov 2017.

[27] Jurka J (1998). Repeats in genomic DNA: mining and meaning. Curr Opin Struct Biol.

[28] Smit AFA, Hubley R, Green P. RepeatMasker Open.4.0. 2013–2015. http://www.repeatmasker.org. Accessed 24 Nov 2017.

[29] Li H, Durbin R (2010). Fast and accurate long-read alignment with Burrows-Wheeler transform. Bioinformatics.

[30] Jun SR, Sims GE, Wu GA, Kim SH (2010). Whole-proteome phylogeny of prokaryotes by feature frequency profiles: an alignment-free method with optimal feature resolution. PNAS.

[31] Zhu YO, Siegal ML, Hall DW, Petrov DA (2014). Precise estimates of mutation rate and spectrum in yeast. PNAS.

[32] Plotkin JB, Kudla G. Synonymous but not the same: the causes and consequences of codon bias. Nat Rev Genet. 2011;12:32–42. 10.1038/nrg2899 PMC3074964 21102527

[33] Lesecque Y, Mouchiroud D, Duret L (2013). GC-biased gene conversion in yeast is specifically associated with crossovers: molecular mechanisms and evolutionary significance. Mol Biol Evol.

[34] Li L, Stoeckert CJ, Roos DS (2003). OrthoMCL: identification of ortholog groups for eukaryotic genomes. Genome Res.

[35] Wang Y, Tang H, Debarry JD, Tan X, Li J, Wang X (2012). MCScanX: a toolkit for detection and evolutionary analysis of gene synteny and collinearity. Nucleic Acids Res.

[36] Hall C, Brachat S, Dietrich FS (2005). Contribution of horizontal gene transfer to the evolution of Saccharomyces cerevisiae. Eukaryot Cell.

[37] Edgar RC (2004). MUSCLE: multiple sequence alignment with high accuracy and high throughput. Nucleic Acids Res.

[38] Kumar S, Stecher G, Tamura K (2016). MEGA7: molecular evolutionary genetics analysis version 7.0 for bigger datasets. Mol Biol Evol.

[39] Field D, Glöckner FO, Garrity GM, Gray T, Sterk P, Cochrane G (2008). Meeting report: the fourth Genomic Standards Consortium (GSC) workshop.

[40] Bartling FG. Ordines naturales plantarum. Gottingae, Sumtibus. 1830.

[41] Cavalier-Smith T (1998). A revised six-kingdom system of life. Biol Rev Camb Philos Soc.

[42] Eriksson OE, Winka K. Supraordinal taxa of Ascomycota. Myconet. 1997;1:1–16.

[43] Kudryavtsev VI (1960). Die Systematik der Hefen.

[44] Zender. 'Pichiacées'. Bull. Soc. bot. Genève. 1925;2 sér. 17:290.

[45] Berkhout CM (1923). De schimmelgeslachten Monilia. Oidium.

[46] Ashburner M, Ball CA, Blake JA, Botstein D, Butler H, Cherry JM (2000). Gene ontology: tool for the unification of biology. Nat Genet.

