# Supplementary figures and images for: Genome overview of eight Candida boidinii strains isolated from human activities and wild environments
Source: Stand Genomic Sci. 2017 Dec 2;12:70. doi: 10.1186/s40793-017-0281-z (PMC5712119; doi:10.1186/s40793-017-0281-z)

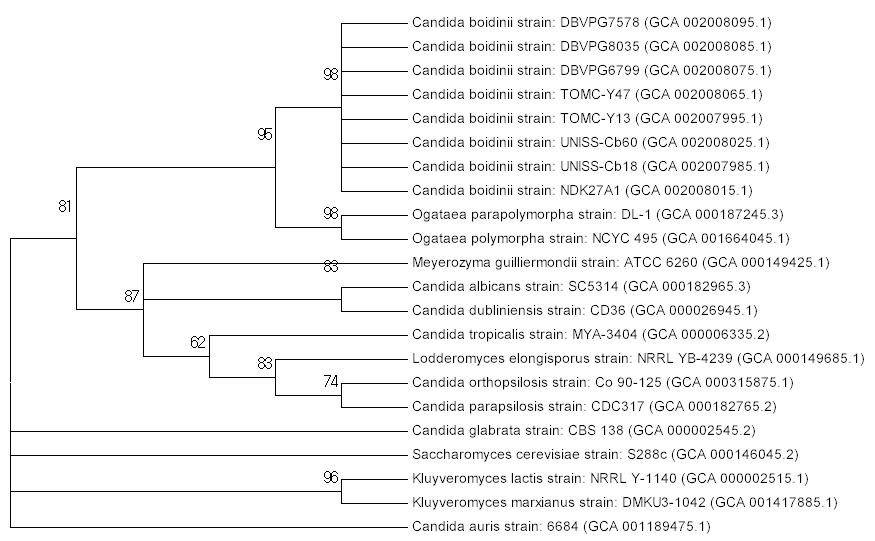

Supplement: Supplementary file 1 — Phylogenetic position of the eight sequenced C. boidinii strains based on D1/D2 domain of 26S rRNA sequences. Genbank assembly accession numbers of the aligned sequences are indicated in brackets. C. boidinii (strain SA18S03) D1/D2 domain (accession id EF460654.1) was used as a query to retrieve the homologues sequences in the other presented species. Low coverage alignment prevented the inclusion of the published C. boidinii strain in the analysis. Sequences were aligned using MUSCLE [37], and the phylogenetic tree was determined using the neighbour-joining algorithm with the Kimura 2-parameter distance model in MEGA (version 7) [38]. A gamma distribution (shape parameter = 1) was used for rate variation among sites. The optimal tree with the sum of branch lengths = 1.5319 is shown, and nodes that appeared in more than 50% of replicate trees in the bootstrap test (1000 replicates) are marked with their bootstrap support values. (TIFF 1387 kb) [file 40793_2017_281_MOESM1_ESM.tif]
