# Supplementary material for: Genome overview of eight Candida boidinii strains isolated from human activities and wild environments
Source: Stand Genomic Sci. 2017 Dec 2;12:70. doi: 10.1186/s40793-017-0281-z (PMC5712119; doi:10.1186/s40793-017-0281-z)
Supplement: Supplementary file 2 — Number of reads generated upon sequencing of eight C. boidinii strains. (DOCX 15 kb) [file 40793_2017_281_MOESM2_ESM.docx]

**Additional file 2: Table S1.** Number of reads generated upon sequencing of eight *C. boidinii* strains (ML = mean length)

|  |  | **Before quality control** | | |  | **After quality control** | | |
| --- | --- | --- | --- | --- | --- | --- | --- | --- |
| **Strain** |  | **# paired end reads** | **R1 ML** | **R2 ML** |  | **# paired end reads** | **R1 ML** | **R2 ML** |
| **UNISS-Cb18** |  | 3,611,910 | 286.1 | 286.6 |  | 3,516,586 | 265.8 | 231.9 |
| **UNISS-Cb60** |  | 3,101,138 | 289.4 | 289.8 |  | 2,992,724 | 270.5 | 231.9 |
| **TOMC-Y13** |  | 2,497,497 | 287.5 | 288.2 |  | 2,415,563 | 267.1 | 226.7 |
| **TOMC-Y47** |  | 2,646,798 | 290.7 | 291.0 |  | 2,544,880 | 268.9 | 228.0 |
| **DBVPG6799** |  | 2,949,331 | 287.2 | 287.8 |  | 2,854,813 | 261.9 | 222.7 |
| **DBVPG7578** |  | 2,839,649 | 288.7 | 288.9 |  | 2,746,973 | 264.9 | 229.5 |
| **DBVPG8035** |  | 3,600,254 | 271.4 | 272.6 |  | 3,527,342 | 256.6 | 227.9 |
| **NDK27A1** |  | 4,377,518 | 286.1 | 286.6 |  | 4,257,495 | 268.8 | 232.0 |
