# Supplementary material for: Genome overview of eight Candida boidinii strains isolated from human activities and wild environments
Source: Stand Genomic Sci. 2017 Dec 2;12:70. doi: 10.1186/s40793-017-0281-z (PMC5712119; doi:10.1186/s40793-017-0281-z)
Supplement: Supplementary file 3 — Number of predicted genes showing high homology (e-value < 0.0001) with gene models predicted in several Candida related species. The data refers to the analysis of strain Cb18 with four different Augustus training sets. (DOCX 14 kb) [file 40793_2017_281_MOESM3_ESM.docx]

**Additional file 3: Table S2.** number of predicted genes for *C. boidinii* strain Cb18 as obtained by the software Augustus with four different training sets.

| **Species** | **Number of predicted genes** | **Number of genes with high homology** |
| --- | --- | --- |
| ***C. tropicalis*** | 5819 | 5404 |
| ***C. Ablcans*** | 5811 | 5377 |
| ***C. glabrata*** | 5636 | 5192 |
| ***C. guilliermondii*** | 3425 | 3118 |
