# Supplementary material for: Genome overview of eight Candida boidinii strains isolated from human activities and wild environments
Source: Stand Genomic Sci. 2017 Dec 2;12:70. doi: 10.1186/s40793-017-0281-z (PMC5712119; doi:10.1186/s40793-017-0281-z)
Supplement: Supplementary file 4 — Number of genomic bases included in transposable elements, simple repeats and low complexity regions of eight C. boidinii strains. (DOCX 14 kb) [file 40793_2017_281_MOESM4_ESM.docx]

**Additional file 4: Table S3.** number of bases included in transposable elements, simple repeats and low complexity regions for eight *C. boidinii* strains.

| **Repeat type** | **UNISS-Cb18** | **UNISS-Cb60** | **DBVPG6799** | **NDK27A1** | **TOMC-Y13** | **TOMC-Y47** | **DBVPG7578** | **DBVPG8035** |
| --- | --- | --- | --- | --- | --- | --- | --- | --- |
| SINE(bp) | 0 | 0 | 0 | 0 | 0 | 0 | 0 | 0 |
| LINE(bp) | 14,179 | 14,131 | 12,298 | 16,408 | 7,266 | 6,862 | 9,814 | 5,322 |
| LTR(bp) | 33,868 | 34,269 | 34,994 | 47,252 | 6,560 | 4,879 | 9,908 | 11,497 |
| DNA elements(bp) | 0 | 0 | 0 | 0 | 0 | 0 | 0 | 0 |
| Unclassified(bp) | 395,079 | 386,476 | 405,109 | 391,601 | 211,950 | 213,794 | 214,881 | 221,110 |
| Simple repeats(bp) | 906,806 | 910,695 | 910,591 | 918,932 | 1,210,818 | 1,214,946 | 1,216,117 | 1,209,594 |
| Low Cimplexity(bp) | 141,377 | 137,773 | 139,150 | 139,350 | 189,308 | 192,009 | 194,746 | 192,027 |
