# Supplementary material for: Genome overview of eight Candida boidinii strains isolated from human activities and wild environments
Source: Stand Genomic Sci. 2017 Dec 2;12:70. doi: 10.1186/s40793-017-0281-z (PMC5712119; doi:10.1186/s40793-017-0281-z)
Supplement: Supplementary file 5 — Alignment statistics for the Blast search of two D1D2 ribosomal portions (isolated and sequenced from one high GC and one low GC content strain) in the eight C. boidinii strains. (DOCX 15 kb) [file 40793_2017_281_MOESM5_ESM.docx]

**Additional file 5: Table S4.** Alignment statistics for the Blast search of two D1/D2 26S ribosomal portions (isolated and sequenced from one high GC and one low GC content strain) in the eight *C. boidinii* strains.

| **Sequence** | **D1/D2** | | |  | **D1/D2** | | |
| --- | --- | --- | --- | --- | --- | --- | --- |
| **Strain** | **DBVPG6799** | | |  | **DBVPG7578** | | |
|  | %Homology | Coverage | e-value |  | %Homology | Coverage | e-value |
| **UNISS-Cb18** | 99.8 | 530 | 0 |  | 100.0 | 529 | 0 |
| **UNISS-Cb60** | 99.8 | 530 | 0 |  | 100.0 | 529 | 0 |
| **DBVPG6799** | 100.0 | 530 | 0 |  | 99.8 | 529 | 0 |
| **NDK27A1** | 100.0 | 167 | 0 |  | 100.0 | 166 | 0 |
| **TOMC-Y13** | 99.8 | 530 | 0 |  | 100.0 | 529 | 0 |
| **TOMC-Y47** | 99.8 | 530 | 0 |  | 99.8 | 530 | 0 |
| **DBVPG7578** | 99.8 | 530 | 0 |  | 100.0 | 529 | 0 |
| **DBVPG8035** | 99.8 | 530 | 0 |  | 100.0 | 529 | 0 |
